# Effect of Sigma Phase Morphology on the Degradation of Properties in a Super Duplex Stainless Steel

**DOI:** 10.3390/ma11060933

**Published:** 2018-06-01

**Authors:** Vahid A. Hosseini, Leif Karlsson, Sten Wessman, Nuria Fuertes

**Affiliations:** 1 Department of Engineering Science, University West, SE-461 86 Trollhättan, Sweden; leif.karlsson@hv.se (L.K.); Sten.wessman@hv.se (S.W.); 2 Innovatum AB., Trollhättan, SE-461 29 Trollhättan, Sweden; 3 Swerea KIMAB AB, P.O. Box 7047, SE-164 40 Kista, Sweden; nuria.fuertes@swerea.se

**Keywords:** duplex stainless steel, microscopy, thermodynamic calculations

## Abstract

Sigma phase is commonly considered to be the most deleterious secondary phase precipitating in duplex stainless steels, as it results in an extreme reduction of corrosion resistance and toughness. Previous studies have mainly focused on the kinetics of sigma phase precipitation and influences on properties and only a few works have studied the morphology of sigma phase and its influences on material properties. Therefore, the influence of sigma phase morphology on the degradation of corrosion resistance and mechanical properties of 2507 super duplex stainless steel (SDSS) was studied after 10 h of arc heat treatment using optical and scanning electron microscopy, electron backscattered diffraction analysis, corrosion testing, and thermodynamic calculations. A stationary arc was applied on the 2507 SDSS disc mounted on a water-cooled chamber, producing a steady-state temperature gradient covering the entire temperature range from room temperature to the melting point. Sigma phase was the major intermetallic precipitating between 630 °C and 1010 °C and its morphology changed from blocky to fine coral-shaped with decreasing aging temperature. At the same time, the average thickness of the precipitates decreased from 2.9 µm to 0.5 µm. The chemical composition of sigma was similar to that predicted by thermodynamic calculations when formed at 800–900 °C, but deviated at higher and lower temperatures. The formation of blocky sigma phase introduced local strain in the bulk of the primary austenite grains. However, the local strain was most pronounced in the secondary austenite grains next to the coral-shaped sigma phase precipitating at lower temperatures. Microstructures with blocky and coral-shaped sigma phase particles were prone to develop microscale cracks and local corrosion, respectively. Local corrosion occurred primarily in ferrite and in secondary austenite, which was predicted by thermodynamic calculations to have a low pitting resistance equivalent. To conclude, the influence of sigma phase morphology on the degradation of properties was summarized in two diagrams as functions of the level of static load and the severity of the corrosive environment.

## 1. Introduction

Super duplex stainless steel (SDSS), with approximately equal fractions of ferrite (δ) and austenite (γ), exhibits excellent performance in highly corrosive environments as well as when high mechanical loads are present. The superior stress corrosion cracking resistance of SDSSs make them applicable where austenitic stainless steels may not fulfill the requirements [1]. In addition, their high pitting resistance, with a pitting resistance equivalent (PREN = % Cr + 3.3% Mo + 16% N) above 40, places them among the high corrosion-resistant stainless steels [2,3]. Their tensile strength is higher than that of austenitic and ferritic stainless steels, making them an option for lightweight structures. However, the outstanding mechanical properties and corrosion resistance of SDSSs are achievable only if appropriate processing procedures are followed [4,5].

The processing and/or application of SDSS is limited at temperatures between 250 °C and 1000 °C due to phase decompositions leading to the formation of sigma phase (σ), chi phase (χ), secondary austenite (γ_2_), R-phase (R), Cr-rich ferrite (α′), nitrides, carbides, G-phase, etc. [6,7,8]. The precipitation of deleterious secondary phases, which are mostly enriched in Cr, Mo, and/or N, decrease the local corrosion resistance around the precipitates and often result in local corrosion attacks [1,9,10]. In addition to the loss of corrosion resistance, the precipitation of secondary phases significantly reduces the mechanical properties of SDSS [6]. Among the secondary phases precipitating in DSS, σ is commonly considered as the most frequent deleterious constituent. This phase is a topologically close-packed brittle intermetallic belonging to the P4_2_/mnm space group, mostly precipitating at temperatures between 600 °C and 1000 °C, and is enriched in Cr and Mo [1]. It seriously degrades the material properties as shown, for example, by Deng et al. [11]. They reported an 80% drop in impact energy and a 25 °C reduction in critical pitting temperature after the precipitation of 5% σ. The σ morphology varies from blocky to coral-shaped, which affects the overall corrosion resistance and mechanical properties of the alloy [12].

Functionally graded materials (FGMs) deliver tailored properties and microstructures varying with location inside a component. Hosseini et al. [13] introduced a novel heat treatment technique to fabricate FGMs that are suitable to study the microstructures forming over a wide range of temperatures. With this method, a stationary tungsten inert gas (TIG) arc creates a steady-state temperature gradient for chosen times ranging from a few seconds to several hours. The microstructures at different aging temperatures are therefore physically simulated in a single sample, which dramatically reduces the material characterization time. The microstructure and phase transformation in arc heat-treated SDSS and weld metal were investigated using automatic hardness testing and electron backscattered diffraction (EBSD) in previous studies [14,15]. In addition, the kinetics of spinodal decomposition in the same material was the subject of another study [16].

The influence of aging on σ nucleation and growth, corrosion behavior, and mechanical properties has been the subject of much research on SDSS [17,18]. Most recently, other aspects of σ such as the influence of surface finish on precipitation kinetics [19], the phase field modeling of precipitation [20], and the effect of σ on mechanical properties [21] have been studied. However, σ morphology and its influence on properties for different aging temperatures have not been well-studied. This study thus aims to investigate σ morphology and its effects on the local misorientation (LMO), hardness, crack propagation, sensitization, and corrosion behavior in order to disclose the possible mechanisms leading to the degradation of heat-affected SDSS. A graded microstructure was generated by 10 h of arc heat treatment of 2507 SDSS and investigated using scanning electron microscopy (SEM), EBSD analysis, microhardness mapping, thermodynamic calculations, sensitization screening testing, and critical pitting temperature testing. Finally, the susceptible microstructures were discussed considering different possible degradation mechanisms such as mechanical cracking and local corrosion attacks.

## 2. Materials and Methods

### 2.1. Material and Heat Treatment

A type 2507 SDSS (EN: 1.4410, UNS S32750) ø 99 mm × 6 mm disc was arc heat-treated. The chemical composition of plates received from Outokumpu Stainless Steel AB is listed in Table 1.

**Figure 1:**
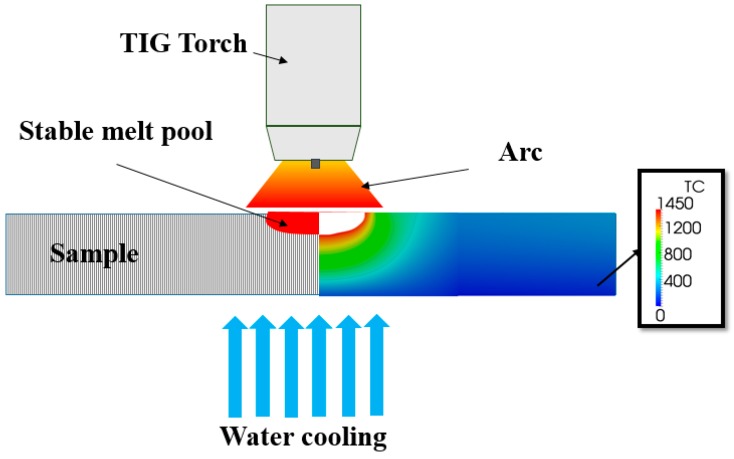
Schematic illustration of arc heat treatment showing a stationary tungsten inert gas (TIG) arc on a super duplex stainless steel (SDSS) disc water-cooled from the backside. A steady-state melt pool and temperature field are formed by the arc heat treatment. The temperature map (right) shows the steady-state temperature distribution in the cross-section.

A novel arc heat treatment method, schematically shown in Figure 1, was used to produce a steady-state temperature gradient in the SDSS disc. A stationary TIG arc was applied to the disc mounted on a water-cooled chamber, producing a stable melt pool and temperature gradient. An arc length of 3 mm, arc current of 100 A, and arc heat treatment time of 10 h were used to produce the functionally graded material. More details about the arc heat treatment and recording of thermal cycles may be found in Reference [13].

### 2.2. Testing and Evaluation

**Figure 2:**
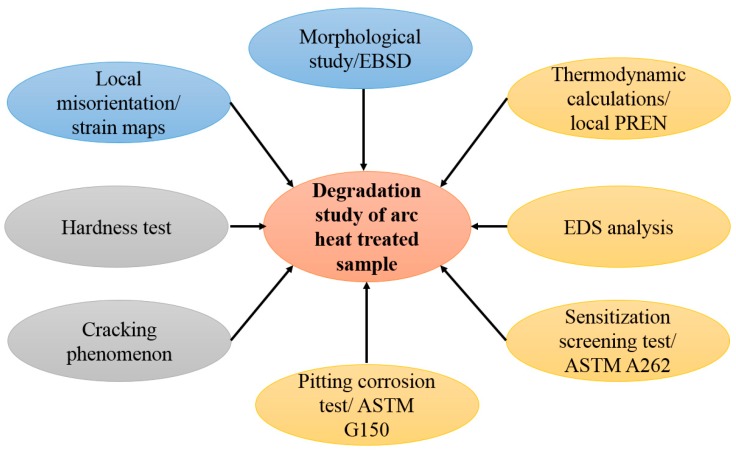
Flowchart of tests and calculations to study the degradation of an arc heat-treated sample. Blue ellipses: Microstructure degradation; gray ellipses: degradation of mechanical properties; orange ellipses: degradation of corrosion resistance.

A flowchart of tests and calculations to study the metallurgical degradation of arc heat-treated 2507 SDSS is shown in Figure 2, where the approaches to investigate microstructure, mechanical properties, and corrosion resistance degradations are displayed with blue, gray, and orange ellipses, respectively.

A cross-section of the arc heat-treated sample was ground and polished using standard procedures followed by polishing with colloidal silica as the final preparation step. The freshly prepared cross-section was used for scanning electron microscopy analyses performed using different microscopes. A Hitachi TM3000 scanning electron microscope (SEM) (Hitachi, Japan) equipped with EDS detector (Hitachi, Japan) was used to measure the Cr, Mo, and Ni content of σ for different heat treatment temperatures. An FEI Magellan SEM (FEI, USA) and a FEI Quanta 650 FEG-SEM (FEI, USA) both equipped with a Nordlys electron backscattered (EBSD) detector (Oxford Instruments, UK) and AZtec 2.2 acquisition software (Oxford Instruments, UK) used for EBSD analysis. Step sizes were chosen between 54 nm and 700 nm and the voltage was 10–20 kV. The HKL Channel 5 post processing software package was used to generate inverse pole figures (IPF) and phase maps. Local phase fractions were determined from the EBSD phase maps.

The same software was employed to measure σ thickness using the intercept method. For each EBSD map, 100 lines were drawn perpendicular to the rolling direction and the distance between points where the lines intersected the sigma phase particle boundaries were measured. In addition, the average and standard deviation were reported.

Local misorientation (LMO) maps and LMO angle distributions were also calculated by HKL Channel 5 software. An LMO map shows the average LMO between a pixel and its neighboring pixels, and allocates the average value to that pixel. The LMO associated with subgrain and grain boundaries (>5°) was excluded and a binning of 3 × 3 pixels was used for low-resolution maps and 11 × 11 pixels for high-resolution maps. Local plastic deformation introduces a change in the local orientation due to the accumulation of dislocations. Consequently, mapping the local misorientation shows where local plastic deformation occurred [22].

In addition to LMO, an estimate of the extent of deformation, “strain” maps, were also calculated from the maximum misorientation between any two points in a grain, along with assigning a maximum misorientation to the center of the grain. Then grains were contoured by a Gaussian filter.

A Struers DuraScan 80 automated hardness tester was used to map the microhardness following the standard [23]. An indenter load of 200 g with a dwell time of 15 s was applied.

Sensitization screening testing was performed by electrolytically etching the cross-section in 7% oxalic acid, applying 2 V for 30 s, inspired by ASTM A262 Practice A [24], and similar to the procedure used by Hosseini et al. [25]. The reason for using a low acid concentration and short etching time was to find the regions most susceptible to local corrosion attack. An Olympus BX60M optical microscope and a Toshiba TM3000 SEM were employed to document corrosion attacks.

The corrosion resistance of the specimen was tested using the procedure described in ASTM G150 [26]. The specimen was immersed in 1 M NaCl at room temperature under a fixed potential of +700 mV saturated calomel electrode (SCE). An SCE and a platinum net were used respectively as reference and auxiliary electrodes. The specimen acted as the working electrode. The temperature of the electrolyte was increased by 1 °C/min starting at room temperature. The critical pitting temperature (CPT) was determined as the temperature at which the current density exceeded 100 μA/cm^2^ for >1 min. After testing, the sample was carefully cleaned and dried with ethanol and the surface was investigated using light optical microscopy (LOM).

### 2.3. Modeling and Calculations

The Open FOAM^®^ software (ESI group, Paris, France) was used to model the temperature distribution in the cross-section during arc heat treatment as presented in detail in References [13,27]. The actual geometry of the fusion boundary was measured from cross-sections of the arc heat-treated sample and steady-state temperatures were recorded by thermocouples to define boundary conditions. The calculated steady-state temperature distribution during arc heat treatment is shown in Figure 1. Thermo-physical properties of the base material were calculated for the actual chemical composition using JMatPro software (Version 6.2.1, Sente Software Ltd., Guildford, UK).

The temperature-location relationship was given by the calculated temperature distribution and the location-microstructure relationship was obtained from the actual cross-section of the arc heat-treated sample. Finally, the temperature-microstructure relationship was found by combining the abovementioned datasets.

Thermodynamic calculations were performed to calculate the chemical compositions of sigma phase and secondary austenite at different temperatures using JMatPro software. The actual chemical composition of the as-received plate (Table 1) was used as input for calculations.

## 3. Results

The arc heat treatment created a steady-state temperature field in the 2507 SDSS disc, maintained for 10 h, which resulted in the formation of a graded microstructure. In this section, the influence of aging temperature on σ morphology, hardness, local plastic deformation, crack propagation, and sensitization behavior in the functionally graded super duplex microstructure is presented.

### 3.1. Microstructure before Arc Heat Treatment

**Figure 3:**
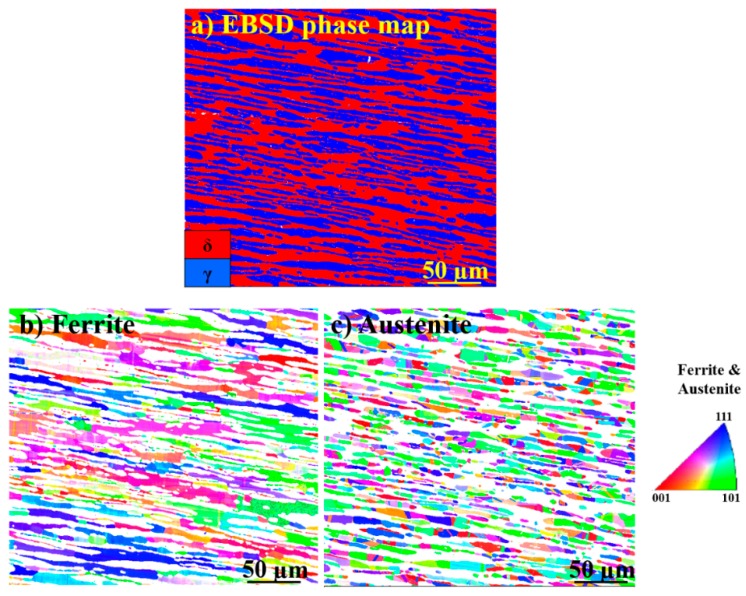
(**a**) Electron backscattered diffraction (EBSD) phase map of the SDSS before arc heat treatment, showing the approximate 50:50 ferrite (δ) to austenite (γ) ratio. No traces of any additional phases were found. (**b**,**c**) Inverse pole figures of δ and γ.

EBSD phase and IPF maps from the SDSS before arc heat treatment are shown in Figure 3. The initial microstructure consisted of balanced δ and γ fractions; no traces of any deleterious secondary phases were found.

Inverse pole figure (IPF) maps of δ and γ in Figure 3 show grains elongated in the rolling direction. The matrix phase is δ. As can be seen in Figure 3c, γ has finer grains compared to ferrite and contains twin boundaries.

### 3.2. Sigma Phase Characteristics in Functionally Graded Microstructure

**Figure 4:**
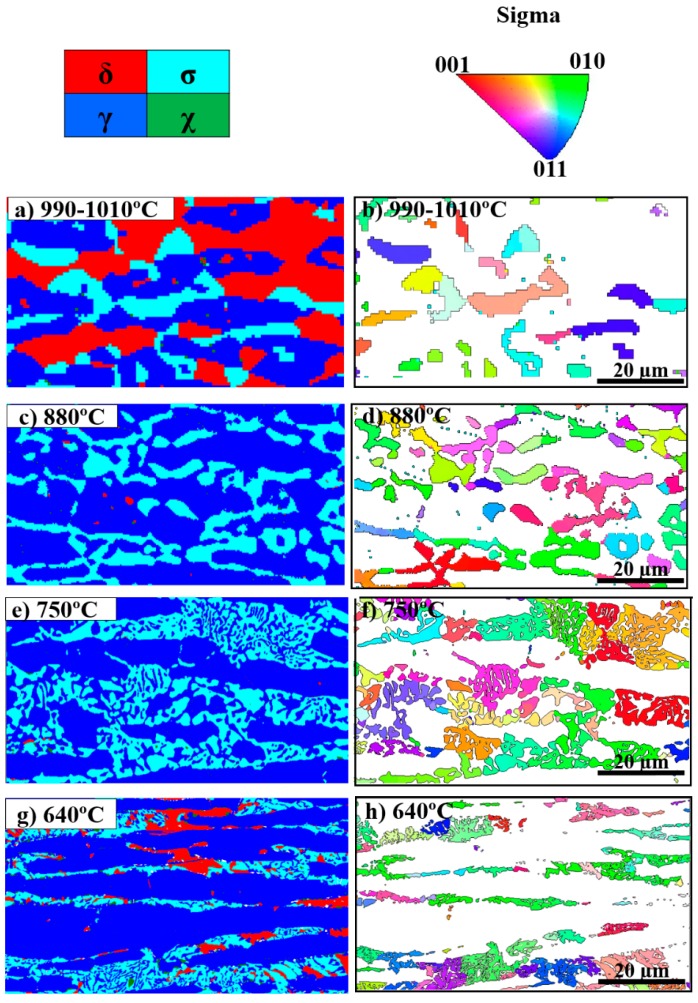
EBSD phase (**left**) and inverse pole figure (IPF) (**right**) maps showing sigma phase (σ) precipitating at different temperatures. The morphology of σ changed from blocky to coral-shaped with decreasing temperature. The phase and pole figure map at (**a**,**b**) 990–1010 °C, (**c**,**d**) 880 °C, (**e**,**f**) 750 °C, and (**g**,**h**) 640 °C.

The sigma phase precipitated between 630 °C and 1010 °C in the graded microstructure. The EBSD phase and IPF maps illustrating the distribution and morphology of σ precipitating at different aging temperatures are given in Figure 4. The microstructure consists mainly of δ, primary γ, γ_2_, σ, and χ; however, other phases such as nitrides and R were also found in the microstructure using a combination of SEM, EBSD, and EDS analyses. Phase fractions of the three major phases in the graded microstructure are given in Table 2. Furthermore, a detailed discussion about the evolution of phase fractions with temperature was presented in Reference [14]. As may be seen, the phase fractions gradually changed with the σ fraction increasing up to 34.4% with decreasing temperature down to 750 °C. It then gradually decreased and no σ was found below 630 °C.

**Figure 5:**
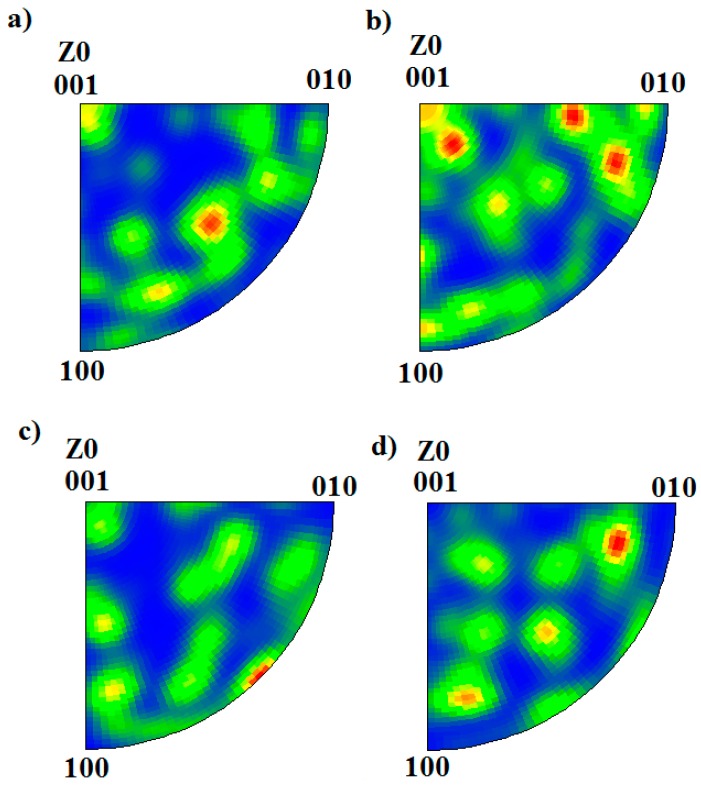
IPF of σ precipitating at (**a**) 990 °C, (**b**) 880 °C, (**c**) 750 °C, and (**d**) 640 °C.

In addition to changes in the σ fractions determined from Figure 4, σ IPF maps are also presented in Figure 4. Sigma phase precipitated as blocky particles at high temperatures, while it became more coral-shaped at lower temperatures. Figure 5 presents the IPF of sigma phase at different temperatures, showing that no specific temperature-dependent texturing was observed.

**Figure 6:**
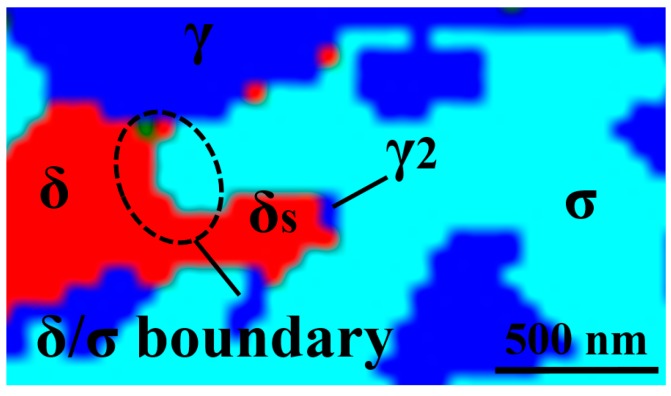
High-magnification EBSD phase map for 640 °C, suggesting that the δ/σ boundary had already passed when γ_2_ started forming.

A high-resolution EBSD phase map at 640 °C is shown in Figure 6. The σ-particle grew inside the ferrite (broken line ellipse), while γ_2_ nucleated further away in ferrite (δs) surrounded by σ branches.

The average thickness of σ for different heat treatment temperatures is listed in Table 2. As can be seen, σ thickness decreased with decreasing temperature, going from 2.9 µm at 990 °C to 0.5 µm at 640 °C, which corresponds to about an 81% reduction in thickness.

The chemical composition of σ precipitating at different aging temperatures is given in Table 3. The σ Mo content significantly decreased from 7.5 wt % to 4.8 wt % when decreasing the temperature from 990 °C to 640 °C. Its Cr and Ni content, on the other hand, remained roughly constant for different aging temperatures.

The hardness was extracted from a microhardness map and representative hardness levels for different heat treatment temperatures are listed in Table 2. The functionality of the graded material, shown by hardness, was significantly affected by changes in σ fractions. The maximum hardness was achieved at 750 °C, where 34% σ precipitated.

### 3.3. Local Misorientation and Strain Distribution Analyses of SDSS FGM

**Figure 7:**
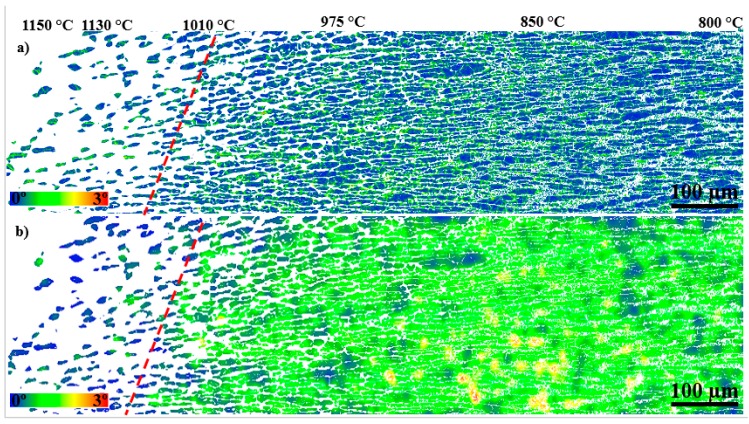
(**a**) A low-resolution austenite local misorientation (γ-LMO) map showing the highest local misorientation in the region where blocky σ precipitated; (**b**) EBSD-estimated “strain” distribution map for austenite. The broken line shows 1010 °C isotherm lines, where σ started precipitating.

**Figure 8:**
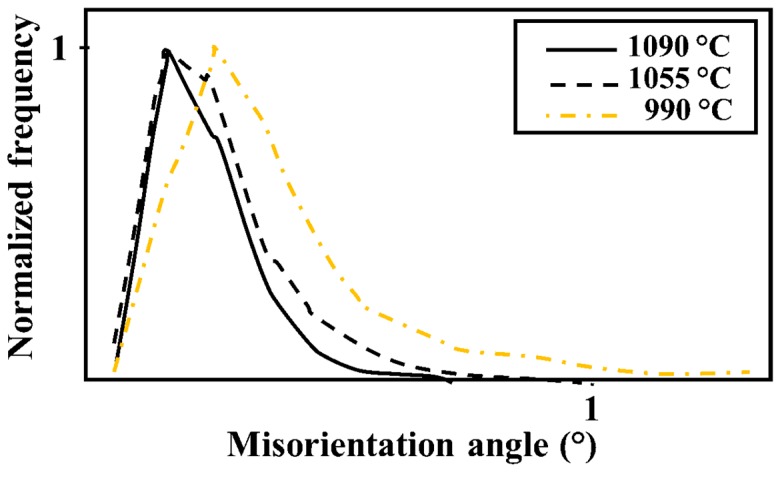
LMO angle distributions for γ at different aging temperatures.

An LMO map for austenite (γ-LMO), for a region heat-treated at temperatures in the range 800–1150 °C, is given in Figure 7a. More green areas, indicating higher γ-LMO, are present in the map next to the σ precipitation start isotherm. As shown in Figure 7a, after a slight increase in γ-LMO at temperatures down to 850 °C, it decreased with decreasing temperature. A similar trend can be seen in the strain map (Figure 7b), where many locations with yellow, orange, and red colors may be found between 850 and 975 °C. The angle γ-LMO distributions extracted from a higher resolution map are shown in Figure 8, illustrating a higher γ-LMO at 990 °C than those at 1090 °C and 1055 °C.

**Figure 9:**
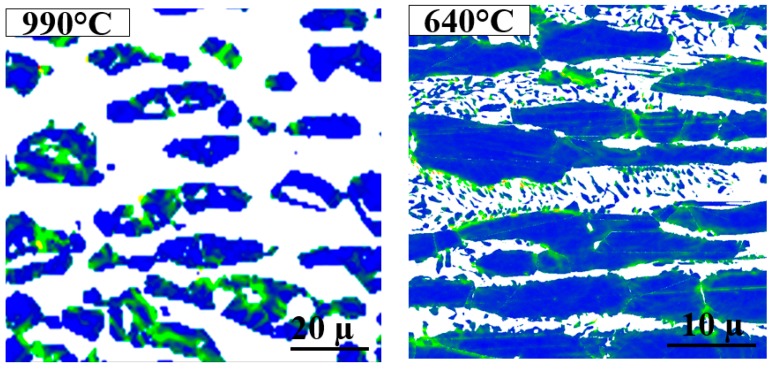
High-resolution γ-LMO maps, where the green areas have higher LMO. γ-LMO is lower in the bulk of primary γ at lower temperatures; however, it is quite high in the γ close to σ/primary γ boundaries.

High-resolution maps of γ-LMO at 990 °C and 640 °C are shown in Figure 9. As explained in Section 2, it is not correct to compare angular γ-LMO distributions at these two temperatures, as the step size used when producing the maps differ. Qualitative analyses of the maps, however, demonstrate that high LMO values are found in the bulk of the primary austenite at 990 °C, but more locally in γ close to σ/γ boundaries at 640 °C.

### 3.4. Cracking in Sigma Phase Formation Zone

**Figure 10:**
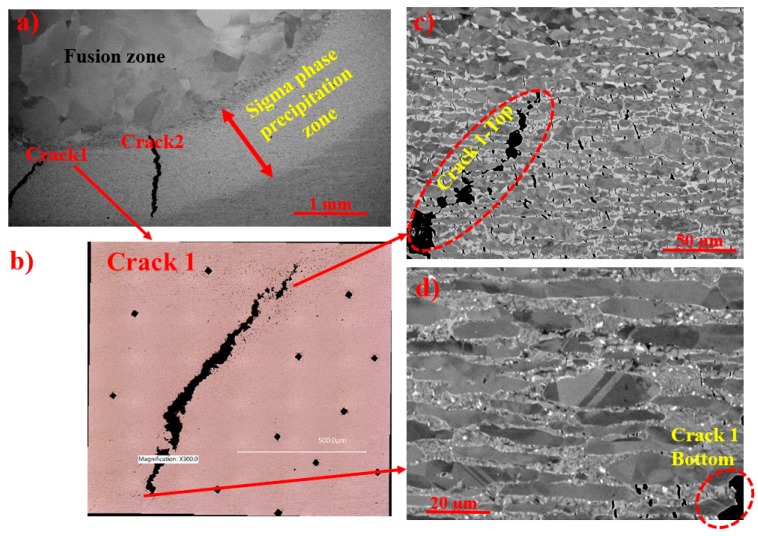
(**a**) Bright etching σ precipitation band, with two macroscale cracks. (**b**) Optical micrograph from Crack 1 surrounded by microscale cracks. Blocky σ (**c**) with numerous larger microscale cracks compared to those in (**d**) coral-shaped σ.

A SEM micrograph from a cross-section of the SDSS disc arc heat-treated for 10 h is shown in Figure 10a. The bright imaging band in the graded microstructure shows where σ precipitated in the temperature range of 630–1010 °C. Two macroscale cracks formed in this zone, marked as Crack 1 and Crack 2. None of the two cracks propagated outside the region where σ precipitated.

Higher magnification SEM micrographs from Crack 1 in Figure 10c,d show that the crack propagated along the σ network. In addition to the macroscale cracks, many microscale cracks formed in σ and were most frequent in blocky σ precipitating at higher temperatures.

### 3.5. Sensitization

**Figure 11:**
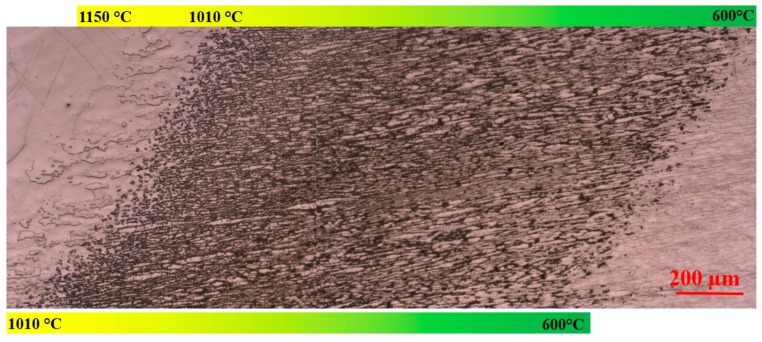
Optical micrograph of cross-section after sensitization screening test by 10% oxalic acid showing sensitization in the σ-containing region. Heat treatment temperatures are shown with color bars.

The sensitization screening test was performed on freshly prepared cross-sections. An example is shown in Figure 11, where the aging temperatures are shown by color bars. It is evident that the σ precipitation band showed indications of sensitization, while regions heat-treated above 1010 °C and below 600 °C did not.

**Figure 12:**
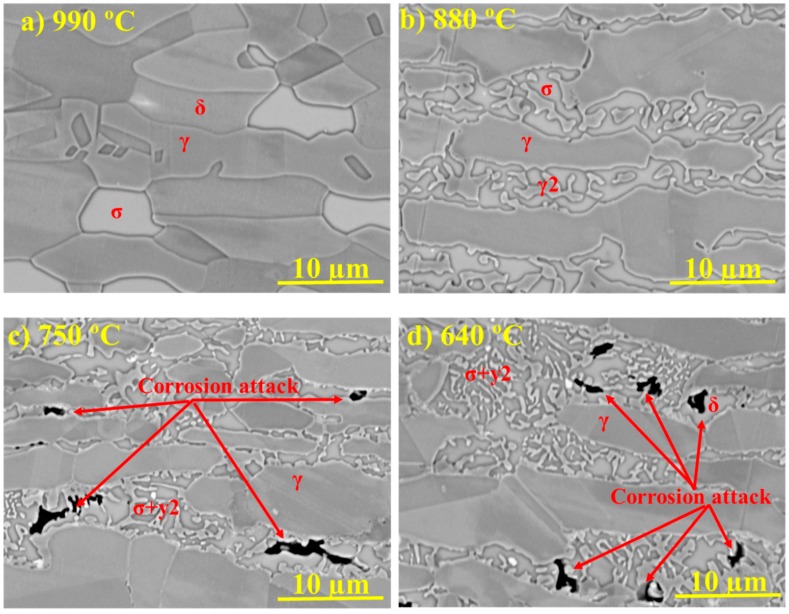
BSE SEM micrographs of SDSS after arc heat treatment at different aging temperatures and sensitization screening testing in oxalic acid: (**a**) 990 °C with very little sensitization, (**b**) 880 °C with some sensitization, and (**c**) 750 °C and (**d**) 640 °C with local corrosion attacks found in δ and/or γ_2_ next to the coral-shaped σ.

SEM micrographs of the graded microstructure for different aging temperatures after the sensitization screening test are presented in Figure 12. The degree of sensitization is most pronounced for heat treatment temperatures between 630 and 750 °C, where coral-shaped σ precipitated. As illustrated in Figure 12 for regions heat-treated at 750 °C and 640 °C, γ_2_ and δ adjacent to the coral-shaped σ seem to be the most susceptible to localized corrosion.

### 3.6. Electrochemical Critical Pitting Temperature Test

**Figure 13:**
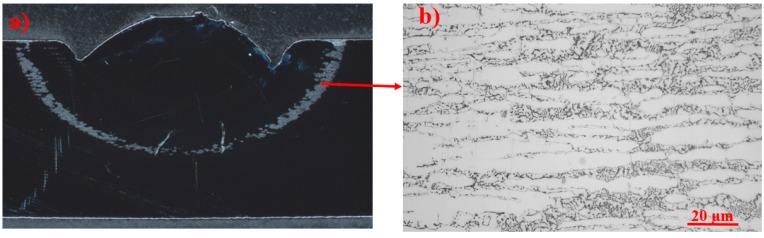
(**a**) Cross-section of the specimen after testing in 1 M NaCl at 700 mV SCE, according to ASTM G150, showing a corroded zone heat-treated at 630–750 °C. (**b**) Optical micrograph of the corroded region showing the attack of secondary γ_2_ and δ next to the coral-shaped σ.

Critical pitting temperature testing according to ASTM G150 was performed to evaluate the local corrosion resistance of the specimen in 1 M NaCl. Pitting occurred at 23.4 °C under an applied potential of 700 mV SCE when the current density exceeded 100 μA/cm². The cross-section and microstructure after the corrosion test are shown in Figure 13, revealing that it was mainly corroded in a region heat-treated at 630–750 °C, leading to the dissolution of δ and γ_2_ around the coral-shaped σ while primary γ was not corroded.

## 4. Discussion

In the present section, first the suitability of the arc heat treatment technique for producing FGM is discussed. Then, the characteristics of σ precipitated at different temperatures and its effects on the corrosion and cracking behavior of σ-containing microstructures are explained. Finally, the influence of different σ morphologies on the degradation of the SDSS FGM from the view of mechanical properties and corrosion resistance is argued.

### 4.1. Use of FGMs for Material Characterization

A functionally graded microstructure, suitable for materials characterization, was produced using the recently introduced arc heat treatment technique [14]. In conventional heat treatment, different initial material conditions (such as residual stress, phase fraction, and segregation) and testing practices (such as sample preparation and etching) may affect the consistency of results. Therefore, more reliable results are expected to be achieved by the characterization of a functionally graded material produced by the arc heat treatment technique, as a single sample is used for the heat treatment for the entire temperature range.

### 4.2. Sigma Phase Characteristics

In a previous study, wavelength-energy dispersive (WDS) measurements showed that nitrogen was depleted in the region close to the fusion boundary heat-treated above 980 °C [14]. This depletion mostly affected the balance between ferrite and austenite but was concluded not to significantly influence σ precipitation temperatures or fractions at lower temperatures [14]. Therefore, the effect of nitrogen loss could be disregarded in the discussion of the present study.

After 10 h of arc heat treatment, σ was the major phase precipitating at temperatures from 630 °C to 1010 °C. In the literature, the δ decomposition to σ + γ_2_ is generally considered as a eutectoid reaction [6,28,29,30]. However, the concept of a eutectoid reaction in duplex stainless steels needs further explanation, as a eutectoid reaction is the transformation of one metastable solid phase into two other stable solid phases. The morphology of eutectoid products such as pearlite in the Fe-Fe_3_C system and some nonferrous alloys has a characteristic layered structure [31] and a classic eutectoid reaction takes place at a constant temperature. The σ precipitation, on the other hand, does not produce the typical eutectoid morphology and occurs over a range of temperatures and can therefore not be considered to be a textbook eutectoid reaction. The σ thickness in individual σ grains varies significantly (Figure 4) and σ morphology does not have a unique fingerprint, particularly where both blocky and coral-shaped σ were found in the same region (Figure 4e,g). In addition, the equilibrium phase diagram does not show typical eutectoid reaction isotherms and the σ fraction varies significantly with temperature [32].

The change in σ morphology from coarse blocky to fine coral-shaped and an 80% reduction in thickness with decreasing precipitation temperature could be explained considering the diffusion and local changes in the chemical composition. The direct transformation of δ to σ, mostly at higher temperatures, is due to the fact that δ can provide Cr and Mo from longer distances by rapid diffusion. Garin et al. [33] also reported direct σ precipitation from δ in cast duplex stainless steel. This condition is suitable for the precipitation of blocky σ occurring at high temperatures. However, very fine ferrite between austenite grains at lower temperatures were also a found to be a possible location for blocky σ, as it is too fine to permit more than one layer of σ to form. It should be mentioned that coarsening can of course also influence the morphology at higher temperatures due to the relatively fast diffusion. The coral-shaped σ precipitating below 800 °C, as also observed in other studies [12], is expected to be the result of a short-range diffusion interaction between growing σ and the parent δ matrix, which results in the precipitation of γ_2_ from locally Cr-depleted δ. This means that γ_2_ may form when a specific chemical composition of ferrite is reached, while the σ/δ phase boundary have already moved away, as shown in a high-magnification EBSD phase map for 640 °C in Figure 6. Hence, the shorter range interaction of δ and σ at lower temperatures leads to the precipitation of a finer, coral-shaped structure. This is in line with the explanation of depleted regions by Sathirachinda et al. [34], in which they clarified that less depletion occurs over a larger distance at higher temperatures and more depletion occurs over a shorter distance at lower temperatures.

Sigma phase did not show any trend of specific texturing at different aging temperatures, as shown in Figure 4 and Figure 5. Ferrite and austenite also did not show any specific texturing before aging, as shown in Figure 3. A similar observation was reported by Warren et al. [35], who concluded that no specific σ nucleation orientation relationship relative to the parent δ was favored. However, it is not possible to conclude whether the apparently random orientation of σ was inherited from the parent δ or not. Therefore, a study on textured δ, such as in weld metal, is needed to investigate its influence on final σ texturing.

**Figure 14:**
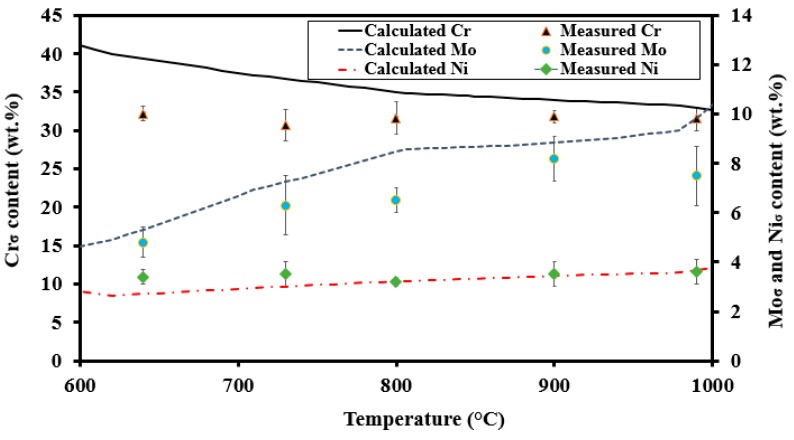
Measured (EDS) and calculated (JMatPro) Cr, Mo, and Ni content of sigma phase precipitated at different temperatures.

A comparison between calculated equilibrium and measured σ compositions is shown in Figure 14. The measured Ni content remained quite constant as also predicted by thermodynamic calculations. The calculations predicted an increase in the σ Cr content with decreasing temperature, but this was not found in the present study. The observed trend of reduction in Mo content with decreasing temperature was well-predicted, although absolute values did not fully agree. Nilsson et al. [36] investigated the chemical composition of σ in a 29Cr-6Ni-2Mo-0.38N alloy and reported a slight reduction in Cr content with decreasing temperature, but a similar trend as that observed in this study for Mo and Ni.

Four temperature ranges are discussed to explain the similarities and differences between the experiments and calculations:

- Above 950 °C, the measured Mo and Cr contents were somewhat lower than predicted. Lower concentrations of Mo and Cr could be a possible consequence of higher σ fractions compared to the Thermo-Calc prediction [14].
- The calculated and measured Mo contents were quite similar around 900 °C. The predicted equilibrium σ content was met and the time was sufficient to approach the equilibrium content.
- Between 750 °C and 850 °C, the Mo content was significantly lower than predicted. Although the calculated equilibrium σ fraction was met at 800 °C [14], the time was not sufficient for the Mo content to approach its equilibrium level.
- Below 750 °C, the Mo content again was close to its predicted equilibrium value, as Mo contents of the parent δ and the final σ were quite similar.

In conclusion, a 10-h heat treatment does not allow the equilibrium chemical composition to be approached, particularly not at temperatures below 850 °C. Results reported by Llorca-Isern et al. [37] indeed showed that the Cr content of σ increased with longer heat treatment time in 2507 SDSS. Lo et al. [38] also reported a 39 wt % Cr content in σ after 10,000 h of aging of UNS 32950 DSS.

### 4.3. Corrosion Behavior of σ-Containing Microstructures

The finer coral-shaped σ was most sensitive to local corrosion (Figure 12 and Figure 13), which is in good agreement with the work of Pohl et al. [12]. A comparison of the present results with those for the solution-annealed 2507 SDSS [39] indicates about a 64 °C reduction in CPT after 10 h of arc heat treatment. The selective dissolution of δ-depleted regions next to sigma and γ_2_ is the caused by the development of local corrosion.

**Figure 15:**
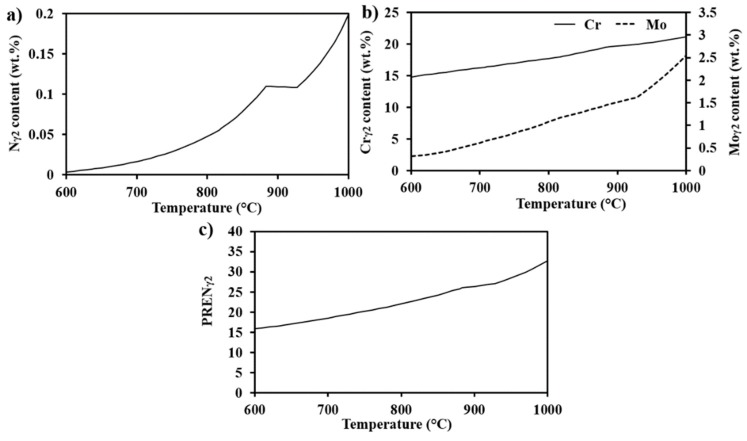
Calculated equilibrium contents of (**a**) N, (**b**) Cr, and Mo in secondary austenite precipitating at different temperatures and (**c**) the resulting PREN.

The more pronounced corrosion attack in σ-containing regions heat-treated at lower temperatures may be explained by thermodynamics and phase transformation kinetics. Corrosion attacks were observed in γ_2_ precipitated next to σ below 750 °C, but not in γ_2_ formed at higher temperatures. PREN calculations of γ_2_ precipitating at different aging temperatures were therefore performed in order to understand this behavior. The equilibrium chemical composition of δ at 1100 °C (solution annealing temperature of the as-received plate) was used as input for calculations of σ + γ_2_ precipitation by the decomposition of δ, and it was assumed that that there was no diffusion of alloying elements between primary γ and γ_2_. The evolution of Mo, Cr, and N contents with the formation temperature and the resulting γ_2_ PREN are shown in Figure 15.

As can be seen in Figure 15a, the nitrogen content of γ_2_ markedly drops with decreasing temperature. The reduction in Cr and Mo content, as shown in Figure 15b, is also significant. The resulting PREN for γ_2_, therefore, decreases from 32 for γ_2_ formed at 1000 °C to 15 when formed at 630 °C (Figure 15c). The predicted decrease of Cr and Mo in γ_2_ is in line with literature [40,41], and together with the lower N content explains the corrosion in γ_2_ precipitating at lower temperatures.

Another possible reason for corrosion of σ-containing microstructures formed at lower temperatures is the expected strong depletion of elements, particularly in the parent matrix δ. At aging temperatures above 900 °C, where δ is stable together with γ and σ, longer heat treatment times allow for a more homogenous distribution of the alloying elements. For example, Yang et al. [42] reported that increasing the aging time from 0.5 h to 5.5 h at 850 °C decreased the corrosion of 2507 SDSS. In contrast, the δ regions next to coral-shaped σ that formed at lower temperatures were sensitized as Cr- and Mo-depleted regions formed (Figure 12 and Figure 13). Similar observations were reported by Magnabosco et al. [29], where both Cr- and Mo-depleted metastable δ were attacked at the earlier aging stage. Hosseini et al. [25] also observed the sensitization of δ in the vicinity of small σ particles in multiply reheated 2507 SDSS. Similarly, even 15 h of aging time at 675 °C did not heal the Cr-depleted region in 2205 DSS [43]. Thermodynamically unstable δ (between 630 and 900 °C) will therefore not be homogenized within practically useable heat treatment times, as it continuously decomposes to secondary phases such as γ_2_ and σ.

### 4.4. Hardness, Local Misorientation, and Crack Propagation

Hardness was clearly correlated to the σ fraction, with a maximum in hardness and the amount of σ at 750 °C (Table 2). Increased hardness can be used as an indicator of embrittlement, but the effect of the σ morphology needs to be studied by other techniques. Investigations of LMO and cracking behavior thus provided more insight into the local degradation of mechanical properties related to different σ morphologies.

The combination of strain distribution, sensitization, and thermodynamic calculation gives an overview of the susceptibility of σ-containing microstructures to degradation. Corrosion resistance has been related to the degree of misorientation in stainless steels, where the higher the angle, the larger the susceptibility to stress corrosion cracking (SCC) and pitting [44,45]. As no external load was applied, it could be concluded that the variation of LMO is a consequence of phenomena occurring during the arc heat treatment. The precipitation of σ increased the γ-LMO (Figure 7 and Figure 8), as also observed by Ubhi et al. [46]. This can be understood in terms of contraction during δ decomposition to γ and σ, as reported by Elmer et al. [47]. The presence of fine γ_2_ together with coral-shaped σ resulted in slightly lower γ-LMO compared to that for blocky σ. However, as calculated by thermodynamic calculations and found by corrosion and sensitization testing, more sensitive γ_2_ precipitated at the lower temperatures. In addition, γ_2_ showed some locally high strain and LMO regions. Therefore, the combination of more sensitive γ_2_ with high local strain can make the microstructure very susceptible to SCC.

For heat treatments at about 850–950 °C, many cracks formed due to the stresses induced by σ precipitation, which could be explained as follows. The arc heat treatment and precipitation of σ and γ_2_ introduced stresses as discussed above. The temperature, phase fractions, and σ morphology varied with distance from the melt pool during arc heat treatment. Hence, the stress distribution was not uniform and the combination of stresses and high temperature processing caused cracking in σ. After some time, the coalescence of microscale cracks led to the formation of macroscale cracks, as shown in Figure 10. Babakr et al. [48] also showed that cracks preferentially follow the σ network in a cast Fe-Cr-Ni. The microstructure containing coral-shaped σ, however, was less susceptible to microscale cracking as the cracks are likely to proceed into the adjacent fine ductile γ_2_, which would stop crack propagation. This is in good agreement with Paul et al. [12], who reported that a microstructure with blocky σ had lower tensile strength than one with coral-shaped σ for similar sigma fractions.

### 4.5. Effects of σ Morphology on Degradation

**Figure 16:**
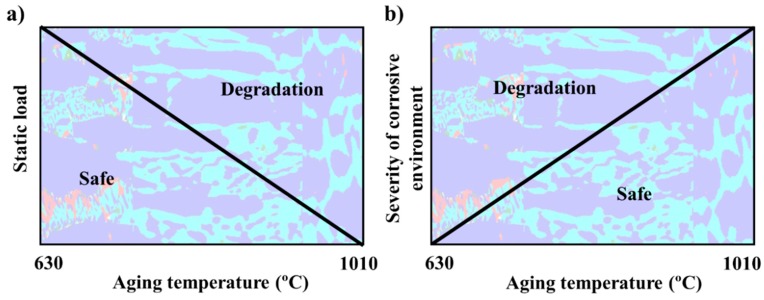
Overview of the degradation of microstructures with different σ morphologies, as a background, and a composite EBSD phase map of σ-containing microstructures is presented. (**a**) Resistance against static external load; (**b**) corrosion resistance.

A schematic overview of the degradation of properties of the heat-treated SDSS as a consequence of resulting σ morphologies for varying mechanical load and corrosive environment is presented in Figure 16. The diagram considers the combination of cracking behavior, local plastic deformation, corrosion, and sensitization screening tests. The diagram is in good agreement with the overall properties of duplex stainless steels with different σ morphologies, where the tensile strength is higher at lower aging temperatures, but general corrosion resistance is lower [12].

The results in this study showed that an accidental increase in operation temperature may lead to loss of mechanical properties and corrosion resistance. Such a temperature increase may also cause the formation of microscale and macroscale cracks, which may prohibit annealing with the aim of retrieving properties. The processing of thick SDSS parts could also be a cause for concern due to uneven heating or cooling.

## 5. Conclusions

The influence of high temperature aging on σ morphology and the resulting degradation of corrosion and mechanical properties was investigated for 2507 super duplex stainless steel. A graded material was produced by a novel heat treatment technique, wherein a stationary arc produced a steady-state temperature gradient for 10 h, covering aging temperatures from ambient to liquidus.

- At temperatures between 630 and 1010 °C, σ precipitated with a maximum fraction of 34% at 750 °C;
- A maximum hardness of about 480 HV0.2 was obtained for aging at about 750 °C;
- The σ morphology changed from blocky to fine coral-shaped with decreasing aging temperature and its thickness decreased by about 80% from 1010 °C to 640 °C. No specific crystallography texturing was found in σ at different aging temperatures;
- The measured chemical composition of σ was similar to that predicted by thermodynamic calculations for 800–900 °C, but deviated at higher and lower temperatures;
- The microstructure containing blocky σ showed a higher local misorientation in the bulk of primary γ grains at 990 °C; however, local misorientation was distributed more locally in the γ_2_ grains next to coral-shaped σ at 640 °C;
- Macroscale cracks were propagated where σ precipitated. Blocky σ particles were more susceptible to microscale cracking;
- Pitting corrosion testing showed that selective corrosion occurred in the regions aged between 630 °C and 750 °C in the microstructure containing fine coral-shaped σ. Sensitization screening testing showed more sensitization at the temperature range of 630–750 °C. Low PREN γ_2_, predicted by thermodynamic calculations, and δ were the location of local corrosion attacks.

## Figures and Tables

**Table 1:** Chemical composition (wt %) of 2507 SDSS, measured with X-ray fluorescence and combustion analysis.

Element	Content (wt %)
C	0.016
Si	0.44
Mn	0.76
P	0.028
S	0.001
Cr	25.04
Ni	6.93
Mo	3.78
N	0.265
Cu	0.40
Fe	Bal.

**Table 2:** Phase fractions, σ thickness and hardness.

T (°C)	δ (%)	γ (%)	σ (%)	Average σ Thickness (µm)	Hardness (HV0.2)
As-received material	49.7 ± 2	50.3 ± 2	0	-	280
1020	82 ± 2	18 ± 2	0	-	277
980–990	36.0–64.3	42.7–28.2	22.3–14.1	2.9 ± 1.7	359
950	-	-	27.0 ± 2.9	-	438
880	0.5 ± 0.2	71.3 ± 1.3	27.9 ± 1.2	1.5 ± 1.1	
750	0.3 ± 0.1	65.0 ± 0.8	34.4 ± 0.7	0.9 ± 0.7	480
640	7.8 ± 3.6	68.3 ± 6.2	21.4 ± 2.7	0.5 ± 0.4	363
630 *	-	-	5.4	-	
620 *	-	-	0	-	

* Only the σ fraction was determined from the SEM micrographs.

**Table 3:** Chemical composition of σ formed at different aging temperatures.

Temperature (°C)	Cr (wt %)	Ni (wt %)	Mo (wt %)	Fe (wt %)
990	31.5 ± 1.5	3.6 ± 0.9	7.5 ± 1.2	Balance
900	31.8 ± 0.8	3.5 ± 0.5	8.2 ± 0.9	Balance
800	31.6 ± 2.1	3.2 ± 0.2	6.5 ± 0.5	Balance
730	30.7 ± 2.0	3.5 ± 0.5	6.3 ± 1.2	Balance
640	32.2 ± 1.0	3.4 ± 0.3	4.8 ± 0.6	Balance
